# Performance of ChatGPT on Ophthalmology-Related Questions Across Various Examination Levels: Observational Study

**DOI:** 10.2196/50842

**Published:** 2024-01-18

**Authors:** Firas Haddad, Joanna S Saade

**Affiliations:** 1 Faculty of Medicine American University of Beirut Beirut Lebanon; 2 Department of Ophthalmology American University of Beirut Medical Center Beirut Lebanon

**Keywords:** ChatGPT, artificial intelligence, AI, board examinations, ophthalmology, testing

## Abstract

**Background:**

ChatGPT and language learning models have gained attention recently for their ability to answer questions on various examinations across various disciplines. The question of whether ChatGPT could be used to aid in medical education is yet to be answered, particularly in the field of ophthalmology.

**Objective:**

The aim of this study is to assess the ability of ChatGPT-3.5 (GPT-3.5) and ChatGPT-4.0 (GPT-4.0) to answer ophthalmology-related questions across different levels of ophthalmology training.

**Methods:**

Questions from the United States Medical Licensing Examination (USMLE) steps 1 (n=44), 2 (n=60), and 3 (n=28) were extracted from AMBOSS, and 248 questions (64 easy, 122 medium, and 62 difficult questions) were extracted from the book, *Ophthalmology Board Review Q&A*, for the Ophthalmic Knowledge Assessment Program and the Board of Ophthalmology (OB) Written Qualifying Examination (WQE). Questions were prompted identically and inputted to GPT-3.5 and GPT-4.0.

**Results:**

GPT-3.5 achieved a total of 55% (n=210) of correct answers, while GPT-4.0 achieved a total of 70% (n=270) of correct answers. GPT-3.5 answered 75% (n=33) of questions correctly in USMLE step 1, 73.33% (n=44) in USMLE step 2, 60.71% (n=17) in USMLE step 3, and 46.77% (n=116) in the OB-WQE. GPT-4.0 answered 70.45% (n=31) of questions correctly in USMLE step 1, 90.32% (n=56) in USMLE step 2, 96.43% (n=27) in USMLE step 3, and 62.90% (n=156) in the OB-WQE. GPT-3.5 performed poorer as examination levels advanced (*P*<.001), while GPT-4.0 performed better on USMLE steps 2 and 3 and worse on USMLE step 1 and the OB-WQE (*P*<.001). The coefficient of correlation (*r*) between ChatGPT answering correctly and human users answering correctly was 0.21 (*P*=.01) for GPT-3.5 as compared to –0.31 (*P*<.001) for GPT-4.0. GPT-3.5 performed similarly across difficulty levels, while GPT-4.0 performed more poorly with an increase in the difficulty level. Both GPT models performed significantly better on certain topics than on others.

**Conclusions:**

ChatGPT is far from being considered a part of mainstream medical education. Future models with higher accuracy are needed for the platform to be effective in medical education.

## Introduction

Recently, advances in artificial intelligence (AI) models, more specifically natural language processing (NLP), led to the development of large language models (LLMs) that have shown remarkable performance on a variety of tasks [1-3]. ChatGPT is among the most popular of these models. It was developed by OpenAI and has had several version updates since its inception. GPT-3.5 was among the earlier versions developed, followed by GPT-4.0, developed on March 15, 2023, as a more robust, concise, and intelligent model. ChatGPT has become quite famous for its outstanding ability to answer questions and assist in many tasks [4].

Medical education relies highly on standardized multiple-choice examinations to test medical students in an objective and consistent way. Ophthalmologists in the United States pass through the United States Medical Licensing Examination (USMLE) steps 1, 2, and 3, the Ophthalmic Knowledge Assessment Program (OKAP), and the Board of Ophthalmology (OB) Written Qualifying Examination (WQE) by the time they become practicing physicians. Undergraduate and graduate medical students rely on different tools available to prepare for these examinations.

One limitation of the current tools for medical education is the lack of personalization. Question banks used today do not tailor their explanations to users; rather, they present one explanation for each question to all its users. ChatGPT and other LLMs, if proven to be accurate in their ability to answer questions, can provide robust explanations to users, and users can then ask specific questions they need further clarification on. This can be very helpful and educational for users as it can tailor to the needs of each user and help them fill specific knowledge gaps they may have. Additionally, the GPT-3.5 model is freely available to everyone, while GPT-4.0 is available at a premium. As such, it is essential to compare these models to assess whether GPT-4.0’s hypothetical increased abilities justify the price of the membership.

The question of how ChatGPT can be integrated for use in medical education has emerged. With the complexity of ophthalmology, the ability of ChatGPT to accurately answer ophthalmology questions could be of significant value to medical students and residents preparing for the USMLE, OKAP, and OB-WQE. It is also important to compare the performance of both GPT-4.0 and GPT-3.5, since GPT-4.0 is marketed as a more intelligent version of its predecessor.

Therefore, the aim of this study is to evaluate the performance of ChatGPT on ophthalmology questions from USMLE steps 1, 2, and 3, the OKAP, and the OB-WQE using both GPT-3.5 and GPT-4.0. We hypothesize that ChatGPT’s responses are comparable to those of human experts in the field, and that GPT-4.0 performs better than GPT-3.5. The results of this study could have implications for the future use of ChatGPT in medical education and training, and for the development of more efficient and effective tools for examination preparation.

## Methods

### Data Sets

Different data sets were used for the different examinations due to the lack of a central service for all examinations. Questions that included pictures or tables were automatically excluded and were not queried on ChatGPT. AMBOSS [5], a question bank and popular resource for the USMLE was used for steps 1, 2, and 3. A total of 44 questions were included for step 1, 60 for step 2, and 28 for step 3. AMBOSS highlights the difficulty of each question and the percentage of people who chose each answer choice. This allowed us to compare the performance of ChatGPT to the general population [5]. For the OKAP and OB-WQE, 248 questions across the different chapters were taken from *Ophthalmology Board Review Q&A* by Glass et al [6].

### Prompt Engineering

The style and the prompt of the questions asked to ChatGPT have been shown to have an impact on the answer given. To standardize the process of asking the questions to ChatGPT, questions were all formatted in the same way on Word (Microsoft Corp). After removing questions with pictures or tables, the questions were formatted in the manner described by Gilson et al [7]. The question stem was consolidated in 1 paragraph, and then each answer choice was placed on a separate line. Furthermore, the answer choices were separated by 2 empty lines from the main question stem; this was done to optimize the accuracy of the results, avoiding any effect the question format may have on ChatGPT’s ability. An example prompt is shown in Textbox 1.

**An example of a prompt (written by the authors).**

Question: What medical discipline deals with conditions of the eye

A. Dermatology

B. Endocrinology

C. Ophthalmology

D. Rheumatology

### Question Input

All questions were input in ChatGPT on March 5, 2023, for GPT-3.5 and April 15, 2023, for GPT-4.0. We then used Excel (Microsoft Corp) spreadsheets to record whether the answer was correct or not, the percentage of users getting the answer correct (if applicable), the difficulty level (if applicable), and the topic (if applicable).

### Data Analysis

Data analysis was conducted using both Python (Python Software Foundation) and Excel. Excel was used to determine the percentage of correct answers. Python (Python Anaconda Spyder 5.3.3) was used to determine the percentage of correct answers by difficulty, test type, and topic. A chi-square test was conducted on Python to determine whether there are any significant differences in answering correctly based on test type and difficulty. Python was also used to compute the coefficient of correlation (and *P* value) between ChatGPT answering correctly and the percentage of users who got the correct answer. Point-biserial was used to compute the correlation between ChatGPT answering questions correctly and humans answering correctly. Other tests included chi-square analysis and the Fisher exact test to investigate relationships between 2 categorical variables (difficulty level, correct or incorrect answers, etc).

### Ethical Considerations

Since this study does not involve any human participants, institutional review board approval is not necessary for the purpose of this study. This study also respects the rights and copyright of the owners of the resources used and has obtained their approval for using the questions without sharing the questions anywhere in the data or paper.

## Results

A total of 380 questions were queried on ChatGPT. The number of questions for each examination were 44 for step 1, 60 for step 2, 28 for step 3, and 248 for the OKAP and OB-WQE. The total percentage of correct answers was 55% (n=210) across all examinations for GPT-3.5, while it was 70% (n=270) for GPT-4.0. Table 1 shows the number and percentage of correct answers for each examination by each GPT model.

Between GPT-3.5 and GPT-4.0, GPT-4.0 performed significantly better on USMLE steps 2 and 3 and the OB-WQE but not on USMLE step 1. While GPT-3.5’s performance decreased with an increase in the examination level (*P*<.001), GPT-4.0 performed better on USMLE steps 2 and 3 and poorer on the OB-WQE and USMLE step 1. The coefficient of correlation (*r*) between ChatGPT answering correctly and the percentage of humans answering correctly on AMBOSS was 0.21 (*P*=.01) for GPT-3.5 and –0.31 (*P*<.001) for GPT-4.0.

Table 2 highlights the percentage of correct questions based on the difficulty level in the AMBOSS questions and in the OB-WQE questions.

Table 3 highlights the performance of both models according to the different topics in the OB-WQE and OKAP questions. Performance for both models was nonrandom, with both models performing better on certain topics such as corneal diseases, pediatrics, retina, ocular oncology, and neuro-ophthalmology.

**Table 1:** Performance of GPT-3.5 and GPT-4.0 on various examinations.

Examination	Correct answers provided by models^a^, n (%)		*P* value
	GPT-3.5	GPT-4.0	
USMLE^b^ step 1	33 (75)	31 (70.45)	.81
USMLE step 2	44 (73.33)	56 (90.32)	.01
USMLE step 3	17 (60.71)	27 (96.43)	.004
OB-WQE^c^	116 (46.77)	156 (62.90)	<.001

^a^*P*<.001 for between-model comparisons in the proportion of correct answers.

^b^USMLE: United States Medical Licensing Examination.

^c^OB-WQE: Board of Ophthalmology Written Qualifying Examination.

**Table 2:** Performance of GPT-3.5 and GPT-4.0 according to different difficulty levels.

GPT-4.0								GPT-3.5						
Board of Ophthalmology difficulty level	Correct answers^a^, n (%)	AMBOSS^b^					Board of Ophthalmology difficulty level			Correct answers^c^, n (%)		AMBOSS^d^		
		Difficulty level	ChatGPT’s performance (correct answers), n (%)	Human performance (correct answers), %							Difficulty level		ChatGPT’s performance (correct answers), n (%)	Human performance (correct answers), %
1	49 (76)	1	19 (100)	83		1			34 (53)		1		14 (88)	83
2	73 (59)	2	43 (91)	68		2			54 (44.26)		2		36 (77)	68
3	35 (56)	3	38 (84)	53		3			28 (45.16)		3		28 (63)	53
N/A^e^	N/A	4	10 (59)	37		N/A			N/A		4		12 (60)	37
N/A	N/A	5	4 (66.67)	26		N/A			N/A		5		3 (50)	26

^a^*P*=.04 on comparing the performance of GPT-4.0 across different difficulty levels.

^b^*P*=.003 on comparing the performance of GPT-4.0 across different difficulty levels.

^c^*P*=.49 on comparing the performance of GPT-3.5 across different difficulty levels.

^d^*P*=.18 on comparing the performance of GPT-3.5 across different difficulty levels.

^e^N/A: not applicable.

**Table 3:** Performance of GPT-3.5 and GPT-4.0 on various included topics.

Category	Correct answers by GPT-4.0^a^, n (%)	Topic	Correct answers by GPT-3.5^b^, n (%)	*P* value
Cornea, external disease, and anterior segment	28 (74)	Cornea, external disease, and anterior segment	25 (66)	.45
Glaucoma	20 (61)	Glaucoma	16 (48)	.32
Lens and cataract	22 (88)	Lens and cataract	8 (32)	<.001^c^
Neuro-ophthalmology	15 (54)	Neuro-ophthalmology	16 (57)	.06
Oculofacial, plastics, and orbit	17 (50)	Oculofacial, plastics, and orbit	10 (29)	.08
Pediatric ophthalmology and strabismus	14 (61)	Pediatric ophthalmology and strabismus	9 (34)	.07
Refractive management and optics	17 (50)	Refractive management and optics	14 (41)	.46
Retina and ocular oncology	24 (73)	Retina and ocular oncology	18 (54)	.12

^a^*P*=.02 for differences in the number of correct answers provided by GPT-4.0 among different categories.

^b^*P*=.03 for differences in the number of correct answers provided by GPT-3.5 among different topics.

^c^Significant at *P*<.05.

## Discussion

### Principal Findings

Our results indicate that GPT-4.0 is superior to GPT-3.5, and that GPT-3.5 has a below-average accuracy in answering questions correctly. The total proportion of correct answers for GPT-3.5 was 55% (n=210), which is considered a poor performance, while that of GPT-4.0 was 70% (n=270), which is an almost average performance [7]. Students typically must achieve 59%-60% of correct answers to pass, and students perform with an average of around 70%-75% on the aforementioned board examinations [7]. It is interesting to note that GPT-3.5’s performance decreased as examination levels increased. This is probably due to the more clinical nature of the examinations. This was not the case for GPT-4.0, which performed best on USMLE steps 2 and 3.

This study investigates the correlation between ChatGPT-3.5 and -4.0 providing a correct answer and the percentage of human users who provided the answer correctly on AMBOSS. For GPT-3.5, a correlation coefficient of 0.21 (*P*=.01) was noted; whereas, this correlation coefficient was –0.31 (*P*<.001) for GPT-4.0. This implies that GPT-4.0 performed better on questions that fewer users answered correctly.

Although our study is limited in that it did not divide the questions into categories such as diagnosis, treatment, basic knowledge, or surgical planning questions. Looking closely at the lens and cataract section in which the model failed (32% of correct answers for GPT-3.5), it was noted that all the correct answers were basic knowledge questions. Surprisingly, an analysis of incorrect answers showed that almost half of the incorrectly answered questions were also basic knowledge questions. For instance, in one of the questions, the model was unable to identify the collagen fiber type in cataract—a piece of information that is widely available on the internet.

On the other hand, GPT-4.0 performed significantly better on basic knowledge questions. One may postulate that since GPT-4.0 was fed a larger database than was GPT-3.5, it has better abilities in answering basic knowledge questions than GPT-3.5. A study by Taloni et al [8] also noted a significant difference in performance between the 2 models in the cataract and anterior segment diseases categories.

It is unclear why it performed so poorly in the lens and cataract section. It could be hypothesized that managing diseases of the lens and cataract may be mostly surgical. This may not have been fed into this language learning model. Furthermore, surgical management requires input from images and videos, which were excluded from our paper and may have caused the drastic difference in performance. Further studies with more questions are needed to answer this question.

Table 2 outlines the percentage of correct answers based on the difficulty level on both models. GPT-4.0 performed poorer on questions with greater difficulties on both AMBOSS and OB-WQE questions, whereas this observation was not significant in GPT-3.5, indicating that it performed almost equally well across difficulty levels. Gilson et al [7] also reported a similar finding for GPT-3.5. Further studies are needed to explain those findings.

This study also examined the proportion of correct answers based on the different topics. Both models performed significantly better on certain topics than others. This is a novel finding not reported in other studies assessing the performance of ChatGPT. It is interesting to further explore this association and why a model would perform on certain topics better than others. It could be hypothesized that questions on topics such as oculoplastic, which rely on surgical techniques and knowledge of aesthetics, may be more difficult for AI models to answer correctly than topics such as oncology and pathology, which rely more on clinical knowledge. Taloni et al [8] reported a better performance of ChatGPT on clinical rather than surgical cases.

The moderate accuracy of ChatGPT-3.5 has been widely replicated in various studies. Gilson et al [7] found accuracies ranging between 42% and 64.4% in USMLE steps 1 and 2 examinations, numbers similar to those noted in this study [7]. The paper also records a decrease in the proportion of correct answers as difficulty level increases, which has been noted in this study as well. Another study by Huh [9] showed that ChatGPT’s performance was significantly lower than that of Korean medical students in a parasitology examination. A letter to the editor of the journal *Resuscitation* revealed that ChatGPT did not reach the passing threshold for the Life Support examination [10]. The cited studies indicate the moderate capabilities of ChatGPT in answering clinically related questions. More studies are needed to show how we can best optimize ChatGPT for medical education. Mihalache et al [11] assessed the performance of ChatGPT on the OKAP and found that it provided 46% correct answers, not unlike the proportion of OB-WQE questions correctly answered by GPT-3.5 in this study. All the aforementioned studies used ChatGPT-3.5 in their analysis. More recent studies have assessed the efficacy of ChatGPT-4.0. A study by Lim et al [12] assessed the performance of GPT-4.0 on myopia-related questions, and the model performed with 80.6% adequate responses, compared to 61.3% for GPT-3.5. Taloni et al [8] assessed the use of ChatGPT-4.0 and ChatGPT-3.5 in the American Academy of Ophthalmology’s self-assessment questions; their study found that GPT-4.0 (82.4% of correct answers) performed better than both humans (75.7% of correct answers) and GPT-3.5 (65.9% of correct answers). The study also assessed the performance of these models across various topics [8]. Similar to our results, Taloni et al [8] found that ChatGPT performed better on ocular oncology and pathology compared to topics such as strabismus and pediatric ophthalmology. To our knowledge, our study is among the first few to assess the abilities of GPT-4.0 in medical examinations across various levels of education and various board examinations.

When reviewing the explanations provided by ChatGPT, it was noted that the model would randomly either explain the provided answer choice or not. It is particularly remarkable to read how it justified the wrong answer choices. More studies are needed to emphasize and assess the answer justifications of the model. Indeed, having solid explanations is essential for it to become a reliable medical education tool.

Our study is unique in that it assesses the capabilities of ChatGPT in answering ophthalmology-related questions in contrast to other studies that assessed its ability to succeed in general examinations such as USMLE steps 1 and 2. Furthermore, this is the first study to assess the ability of ChatGPT to answer questions of a certain discipline across all its examination levels. Finally, this is among the first studies to compare GPT-4.0’s performance to GPT-3.5’s performance in medical examinations.

ChatGPT can be a great add-on to mainstream resources to study for board examinations. There have been reports of using it to generate clinical vignettes and board examination–like questions, which can create more unique practice opportunities for students. Additionally, our study also assesses the accuracy of the 2 models on board examination questions related to ophthalmology. Students can input questions they need help with on the platform, and receive an answer and explanation by using the platform. If the student is not satisfied with the answer provided, or has further questions, he or she can respond to the model and receive a more personalized answer. This is crucial as it significantly decreases the time needed to study and also creates a tailored study experience for each student’s needs.

However, ChatGPT needs further optimization before it can be considered a mainstream tool for medical education. The image feature was not present in GPT-3.5 and was introduced in GPT-4.0. This feature is available only on demand and is yet to be available to all users. Its accuracy and reliability are yet to be established for examination purposes. Many questions were excluded due to them containing images, which is a considerable limitation considering the visual nature of ophthalmology. Even in the text-only questions, ChatGPT had moderate accuracy in answering questions across different difficulties and levels. This study is, however, limited by the small number of questions, particularly in the USMLE steps, due to the absence of a large number of ophthalmology questions in the resources used to prepare for these examinations. More studies are needed, which input a larger number of questions. This study also does not assess the repeatability of ChatGPT’s answers; however, a study by Antaki et al [13] reported near-perfect repeatability.

### Conclusions

Overall, this study suggests that ChatGPT has moderate accuracy in answering questions. Its accuracy decreases in nature as the examinations become more advanced and more clinical in nature. In its current state, ChatGPT does not seem to be the ideal medium for medical education and preparation for board examinations. Future models with more robust capabilities may soon become part of mainstream medical education. More studies are needed, which input a larger number of questions to verify the results of this study and attempt to find explanations for many of the intriguing findings.

## Abbreviations

AI: artificial intelligence
LLM: large language model
NLP: natural language processing
OB: Board of Ophthalmology
OKAP: Ophthalmic Knowledge Assessment Program
USMLE: United States Medical Licensing Examination
WQE: Written Qualifying Examination
